# The Effect of Human Mesenchymal Stem Cells Derived from Wharton’s Jelly in Spinal Cord Injury Treatment Is Dose-Dependent and Can Be Facilitated by Repeated Application

**DOI:** 10.3390/ijms19051503

**Published:** 2018-05-17

**Authors:** Petr Krupa, Irena Vackova, Jiri Ruzicka, Kristyna Zaviskova, Jana Dubisova, Zuzana Koci, Karolina Turnovcova, Lucia Machova Urdzikova, Sarka Kubinova, Svatopluk Rehak, Pavla Jendelova

**Affiliations:** 1 Department of Neurosurgery, Charles University, Medical Faculty and University Hospital Hradec Králové, Sokolska 581, 50005 Hradec Kralove, Czech Republic; petr.krupa@fnhk.cz (P.K.); rehak@lfhk.cuni.cz (S.R.); 2 Institute of Experimental Medicine, Czech Academy of Sciences, Vídeňská 1083, 14220 Prague 4, Czech Republic; irena.vackova@biomed.cas.cz (I.V.); j.ruzicka@biomed.cas.cz (J.R.); kristyna.zaviskova@biomed.cas.cz (K.Z.); jana.dubisova@biomed.cas.cz (J.D.); zuzana.koci@biomed.cas.cz (Z.K.); karolina.turnovcova@biomed.cas.cz (K.T.); urdzikl@biomed.cas.cz (L.M.U.); sarka.k@biomed.cas.cz (S.K.); 3 Department of Neuroscience, Charles University, Second Faculty of Medicine, 15006 Prague 5, Czech Republic

**Keywords:** spinal cord injury, human mesenchymal stem cells, Wharton’s jelly, inflammatory response, neuroregeneration, astrogliosis, axonal growth

## Abstract

Human mesenchymal stem cells derived from Wharton’s jelly (WJ-MSCs) were used for the treatment of the ischemic-compression model of spinal cord injury in rats. To assess the effectivity of the treatment, different dosages (0.5 or 1.5 million cells) and repeated applications were compared. Cells or saline were applied intrathecally by lumbar puncture for one week only, or in three consecutive weeks after injury. Rats were assessed for locomotor skills (BBB, rotarod, flat beam) for 9 weeks. Spinal cord tissue was morphometrically analyzed for axonal sprouting, sparing of gray and white matter and astrogliosis. Endogenous gene expression (*Gfap*, *Casp3*, *Irf5*, *Cd86*, *Mrc1*, *Cd163*) was studied with quantitative Real-time polymerase chain reaction (qRT PCR). Significant recovery of functional outcome was observed in all of the treated groups except for the single application of the lowest number of cells. Histochemical analyses revealed a gradually increasing effect of grafted cells, resulting in a significant increase in the number of GAP43+ fibers, a higher amount of spared gray matter and reduced astrogliosis. mRNA expression of macrophage markers and apoptosis was downregulated after the repeated application of 1.5 million cells. We conclude that the effect of hWJ-MSCs on spinal cord regeneration is dose-dependent and potentiated by repeated application.

## 1. Introduction

Spinal cord injury (SCI) is a serious mutilating injury, resulting in loss of motor, sensory, and autonomic functions, and remains a challenging medical and social problem even in the 21st century. The final neurological deficit is determined by two mechanisms—primary and secondary injury. Primary injury represents the mechanism and strength of the direct trauma. Secondary injury is characterized by the local immune reaction followed by apoptosis of the injured and vulnerable neurons, tissue atrophy with cavitation, and glial scar formation [1]. Since there is no specific treatment for primary injury, and the endogenous potential to regenerate the spinal cord neurons is very limited [2], several treatments are focused on neuroprotection and/or reducing the impact of secondary pathological processes. Promising results in alleviation of the pathological chain of secondary damage were found in the last decade by application of mesenchymal stem cells (MSCs).

MSCs are multipotent cells with multi-differentiation and self-renewal capacity. Many types of adult and embryogenic tissue have been proven as a source of MSCs—adipose tissue, peripheral blood, lung, heart, corneal stroma, dental pulp, placenta, endometrium, amniotic membrane, and umbilical cord blood and tissue (Wharton’s jelly). Under special conditions in vitro, they are capable of differentiating into various tissue cells such as osteocytes or osteoblasts, adipocytes, and chondrocytes [3,4,5]. However, the regenerative potential of MSCs as a therapeutic tool can be provided mainly by the paracrine effect, interacting with the environment around the injured tissue. In nervous tissue repair and regeneration, MSCs support revascularization, modulate inflammatory response [6], produce different growth factors and cytokines [7], and protect vulnerable cells from oxidative stress, causing stress-induced apoptosis [8]. Therefore, it was recently suggested by Caplan [9] that they should be renamed to medicinal signaling cells (MSCs).

In the current study, we used MSCs isolated from human Wharton’s jelly (WJ-MSCs). Wharton’s jelly is the primitive gelatinous connective tissue of the umbilical cord, first described by Thomas Wharton in 1656. Compared to other sources of MSCs (bone marrow, adipose tissue), WJ-MSCs are more primitive with higher proliferating potential [10], have lower immunogenicity because of a lower major histocompatibility complex class I (MHC-I) and the absence of major histocompatibility complex class II (MHC-II) expression [11], and are proven to be non-tumorigenic [12]. Furthermore, WJ-MSCs can be easily and non-invasively obtained from discarded umbilical tissue, and represent neither potential danger for the donor, nor ethical concerns [13]. They are highly proliferative and can be easily expanded.

The delivery route of stem cells is a frequently discussed issue, as the mode of delivery is an important factor in translation to clinical practice. Therefore, several studies have compared the intraspinal, intrathecal (or intracisternal), intraarterial and intravenous application of MSCs. Regardless of the delivery route, MSC treatment has improved functional recovery after SCI. Whilst the engraftment of the transplanted cells was higher in animals with intraspinal delivery [14,15], injections into the parenchyma of the spinal cord may further damage the spinal cord tissue; therefore, less invasive methods, such as intrathecal or intravenous delivery, are preferable. Indeed, the cells grafted via lumbar puncture, or intracisternally have shown the best functional recovery, even though they often remained in the intrathecal space [16,17] or accumulated around the anterior spinal artery [18]. Rats intrathecally grafted with human bone marrow MSCs (hBM-MSCs) had reduced inflammatory reactions and apoptosis, improved functional recovery, and the glial scar formation had been rearranged after SCI, though no cells were detected in the spinal cord parenchyma two months after transplantation [19,20]. Because of the relatively short survival time of MSCs in the host tissue the repetitive delivery of MSCs may prolong the beneficial effects induced by MSC application by potentiating their regenerative potential [18].

The main goal of this study was to assess the dose of the applied WJ-MSCs, and the difference between single and repetitive application on functional recovery and tissue repair after SCI in rats. WJ-MSCs were applied intrathecally by a lumbar puncture into rats with balloon-induced spinal cord compression lesion. This model simulates the compression of the human spinal cord by an unreduced dislocation or a fracture dislocation of the spine. It is assumed that both mechanical and vascular factors are involved in the pathogenesis of spinal cord injury in this model. Moreover, it is simple and reproducible and requires minimal surgical preparation of the animal (no laminectomy) [21]. The functional outcome was assessed using a set of behavioral tests (BBB, flat beam test, and rotarod). Furthermore, histological and immunohistochemical analyses were performed to evaluate the sparing of gray and white matter, axonal sprouting, and formation of glial scar. Finally, quantitative PCR analyses of selected endogenous genes were performed.

## 2. Results

### 2.1. Cell Culture

hWJ-MSCs phenotype, their multipotent differentiation potential, and high proliferation capacity was evaluated prior to transplantation (see Supplement 1, Figures S1–S3).

### 2.2. Behavioral Analysis

#### 2.2.1. BBB Test

**Figure 1:**
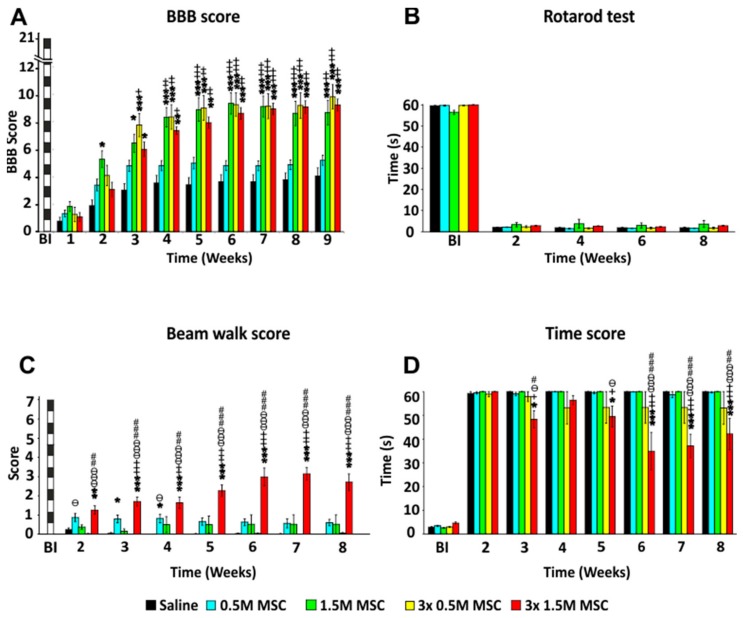
Recovery of locomotor functions following hWJ-MSCs transplantation after SCI. Treatments by a different number of administrated cells. The locomotor skills of saline- or stem cell-treated rats were measured using the BBB (**A**), rotarod (**B**), beam walk score (**C**), and time score (**D**). Animals treated with a higher single dose and by repetitive dose of hWJ-MSCs achieved significantly higher scores in the open-field BBB test when compared to saline controls and animals treated by 0.5 M hWJ-MSCs (**A**). Strength and limb coordination was measured by rotarod test (**B**), where no significant differences were found. The flat beam test (**C**), which is focused on advanced locomotor skills, demonstrated significantly higher scores in the group treated by 3 × 1.5 M hWJ-MSCs. Time score (**D**) reflects the time the rat needs to cross the beam and shows the overall stability of the rat. Animals treated by 3 × 1.5 M achieved significantly better times than the rest of the rats. * *p*  <  0.05 versus saline; ** *p*  <  0.01 versus saline; *** *p*  <  0.001 versus saline; + *p*  <  0.05 versus 0.5 M MSCs; ++ *p*  <  0.01 versus 0.5 M MSCs; +++ *p*  <  0.001 versus 0.5 M MSCs; # *p*  <  0.05 versus 3 × 0.5 M MSCs; ## *p* <  0.01 versus 3 × 0.5 M MSCs; ### *p*  <  0.001 versus 3 × 0.5 M MSCs; ѳ *p*  <  0.05 versus 1.5 M MSCs; ѳѳ *p*  <  0.01 versus 1.5 M MSCs; ѳѳѳ *p*  <  0.001 versus 1.5 M MSCs. BBB = Basso, Beattie, and Bresnahan test; BI = before injury; MSC = human Wharton Jelly mesenchymal stem cell; SCI = spinal cord injury.

Recovery of the hind limb locomotor function was evaluated every week starting the first week after SCI. The BBB score was calculated as a mean value from the scores of both legs (Figure 1A). One week after SCI, all tested animals had severe paraparesis or paraplegia. The average score was 1.28 ± 0.15. No differences between the groups were observed. The second week after SCI animals in all groups showed new motions in one or two joints. Among the groups, a significant difference was found between the control group (2.1 ± 0.43) and the group treated with a single dose of 1.5 M hWJ-MSCs (5.28 ± 0.59) (*p* < 0.05). In the third week after SCI, a rapid improvement of the locomotor functions was observed in the groups treated with a single dose of 1.5 M, and the groups with repeated treatment of 0.5 and 1.5 M, respectively. All three groups recovered significantly better than the control group (*p* < 0.05, *p* < 0.001). Animals treated with only 0.5 M showed little improvement over the control group, which was not statistically significant. In the following weeks, improvement of movement and strength of the hind limbs continued but not so rapidly as was seen during the first three weeks. From the fourth week onward, until the end of the experiment, rats treated with 1.5 M and 3 × 0.5 M and 3 × 1.5 M had comparable results, which were significantly better than the control group and animals treated by 0.5 M (*p* < 0.01, *p* < 0.001). No significant difference between the control group and the 0.5 M group was found. The final results at the end of the ninth week were 4.29 ± 0.57 for the control group, 5.19 ± 0.36 for 0.5 M, 9.81 ± 0.88 for 3 × 0.5 M, 8.67 ± 0.88 for 1.5 M and 9.21 ± 0.45 for 3 × 1.5 M. Animals in the groups with repetitive treatment were mostly able to achieve effective weight support of their body when standing, or even walking. The gap between scores 8 (and lower) and 9 (and higher) is substantial when comparing the strength of the muscles of the hindlimbs.

(Two-way RM ANOVA, Treatment; F = 8.481, *p* ˂ 0.001; for *p* values of post hoc pair-to-pair test, see Supplement 2).

#### 2.2.2. Rotarod Test

Coordination of the limb movements was tested by a rotarod test (Figure 1B). All animals were trained in this task before surgery at a fixed speed of 10 rpm. Testing was then performed every two weeks—2nd, 4th, 6th, and 8th week after SCI. Due to the severity of the lesion and limited recovery, no significant differences were observed between the groups.

(Two-way RM ANOVA, Treatment; F = 0.490, *p* = 0.743; for *p* values of post hoc pair-to-pair test, see Supplement 2).

#### 2.2.3. Beam Walk Test

Advanced locomotor skills and coordination of the hind limbs was measured by walking on the flat beam. Results were evaluated using a 0–7 scale modified from Metz and Whishaw. All animals were firstly trained in this task before surgery and then tested every week starting the second week after SCI (Figure 1C). Due to the severity of the lesion, most of the rats showed minimal ability to cross the beam and often just stayed and balanced at the starting point of the beam. The best scores (7th week after SCI 3.1 ± 0.33) were obtained in animals treated by 3 × 1.5 M, which were significantly better, when compared to all of the other groups. For the first three weeks, animals treated with 0.5 M achieved significantly better results than the control group, but in subsequent weeks they gradually worsened and the significant difference was lost. This was most probably due to the gaining of weight, lack of motivation, and fear of falling off the beam.

(Two-way RM ANOVA, Treatment; F = 20.656, *p* ˂ 0.001; for *p* values of post hoc pair-to-pair test, see Supplement 2).

In addition to the beam score, we measured the time needed to cross the beam (maximally for 60 s). During the pre-training, healthy rats were able to cross the beam in approximately 3.0 s (Figure 1D). The measurement was performed weekly starting the second week after SCI. Two weeks after SCI not all the rats were able to cross the beam and did not move from the starting line. In the following weeks, a significant improvement was observed between the rats treated by 3 × 1.5 M and the other groups. The best score was achieved in the sixth week in the group 3 × 1.5 M, when rats traversed the beam at an average time of 34.9 ± 7 s.

(Two-way RM ANOVA, Treatment; F = 5.001, *p* = 0.002; for *p* values of post hoc pair-to-pair test, see Supplement 2).

### 2.3. Histology and Immunohistochemistry

#### 2.3.1. Gray and White Matter Sparing

**Figure 2:**
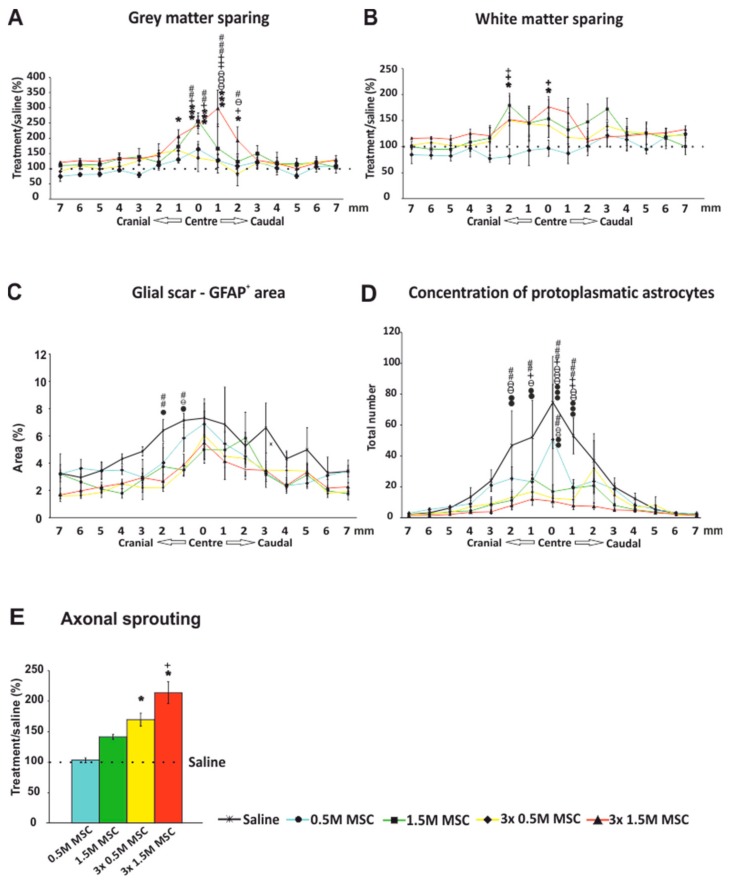
Immunohistochemical and histological analysis 9 weeks after SCI. The total area of spared gray matter was significantly higher in the group treated by 3 × 1.5 M hWJ-MSCs, mainly in the slices near the center of the lesion (**A**). Analysis of the white matter sparing showed similar results to the gray matter, resulting in mild yet still significant preservation in the group treated by 3 × 1.5 M hWJ-MSCs (**B**). The GFAP-CY3 positive area showing the glial scar formation around the central cavity was slightly smaller in all treated groups compared to the control, with a significant difference near the center of the lesion against the groups treated by repetitive doses (**C**). The average number of protoplasmic astrocytes near the center of the lesion was significantly higher in the control group than in the cell-treated groups (**D**). The average number (15 slices per rat; 5 rats) of GAP43+ fibers presented as relative when compared to the control, which is set as 100%. A gradually significant increase with the total number of applied MSCs is shown (**E**). The dotted line represents the value of saline-treated rats (100%). * *p*  <  0.05 versus saline; *** *p*  <  0.001 versus saline; + *p*  <  0.05 versus 0.5 M MSCs; ++ *p*  <  0.01 versus 0.5 M MSCs; +++ *p*  <  0.001 versus 0.5 M MSCs; # *p*  <  0.05 versus 3 × 0.5 M MSCs; ## *p*  <  0.01 versus 3 × 0.5 M MSCs; ### *p*  <  0.001 versus 3 × 0.5 M MSCs; ѳ *p*  <  0.05 versus 1.5 M MSCs; ѳѳ *p*  <  0.01 versus 1.5 M MSCs; ѳѳѳ *p*  <  0.001 versus 1.5 M MSCs; • *p*  <  0.05 versus 3 × 1.5 M MSCs; •• *p*  <  0.01 versus 3 × 1.5 M MSCs; ••• *p*  <  0.001 versus 3 × 1.5 M MSCs. MSCs = human Wharton Jelly mesenchymal stem cells; SCI = spinal cord injury; GFAP-CY3 = glial fibrillary acidic protein cyanine 3; GAP43 = growth-associated protein 43.

The total area of spared gray/white matter was measured on the 15 cross sections of the spinal cord 9 weeks after the SCI (7 sections cranially and caudally to the center of the lesion, which was determined as the section with the smallest area of the residual spinal cord tissue). Values were averaged and compared to the control, which was set as 100%. Concerning the gray matter preservation (Figure 2A), a significant difference was found between the group 3 × 1.5 M and the control (*p* < 0.05), and a strong trend was observed when compared to the group of 0.5 M (*p* = 0.051). Comparison of the white matter sparing showed no significant difference between the groups as a whole (Figure 2B). Similarly to the gray matter, in the center of the lesion and in the surrounding tissue, we observed significantly more spared white matter in animals treated with 3 × 1.5 M and 1.5 M when compared to the control group and rats treated with 0.5 M.

(Two-way RM ANOVA, Treatment; gray matter − F = 3.341, *p* = 0.03, white matter − F = 1.290, *p* = 0.307; for *p* values of post hoc pair-to-pair test, see Supplement 3).

#### 2.3.2. Astrogliosis and Distribution of Protoplasmic Astrocytes

The total area of the glial scar formed around the central cavity was measured on 15 GFAP-CY3 stained cross sections of the spinal cord 9 weeks after the SCI (7 sections cranially and caudally to the center of the lesion, which was determined as the section with the smallest area of the residual spinal cord tissue). Values were averaged and are presented as a ratio of scar tissue to the whole section in percentages (Figure 2C). Groups treated with 3 × 1.5 M as well as 3 × 0.5 M and 1.5 M had a significantly smaller GFAP positive area around the main cavity compared to the control group (*p* < 0.05). The group treated by 0.5 M showed no significant difference compared to saline-treated rats.

(Two-way RM ANOVA, Treatment; F = 4.026, *p* = 0.015; for *p* values of post hoc pair-to-pair test, see Supplement 3).

On the same slices, the number of protoplasmic astrocytes was counted (Figure 2D). Rats treated by 3 × 0.5 M and 1.5 M as well as 3 × 1.5 M had a significantly lower number of protoplasmic astrocytes compared to the control group.

(Two-way RM ANOVA, Treatment; F = 3.997, *p* = 0.015; for *p* values of post hoc pair-to-pair test, see Supplement 3).

#### 2.3.3. Axonal Sprouting

Axonal sprouting was determined as the number of GAP43^+^ fibers, which were manually counted on the 15 cross sections of the spinal cord 9 weeks after the SCI (7 sections cranially and caudally to the center of the lesion, which was determined as the section with the smallest area of the residual spinal cord tissue). Values were averaged and compared to the control, which was set as 100%. The significant effect of the cell treatment was not only dose-dependent, but further improved after repeated application (Figure 2E). Treatment with the lowest dose—0.5 M, had no or minimal effect on axonal sprouting (102 ± 4%). In the other cell-treated groups, the number of positive fibers was gradually increasing with the higher number of grafted cells (1.5 M 140 ± 4%), and repeated application had a significantly stronger effect than a single dose (3 × 0.5 M 168 ± 10%) and (3 × 1.5 M 212 ± 18%) (*p* < 0.05).

(One-way ANOVA, Treatment; H_4_ = 21.844, *p* ˂ 0.001).

### 2.4. qRT-PCR

#### Expression of Intrinsic Genes after Stem Cell Transplantation, after SCI

**Figure 3:**
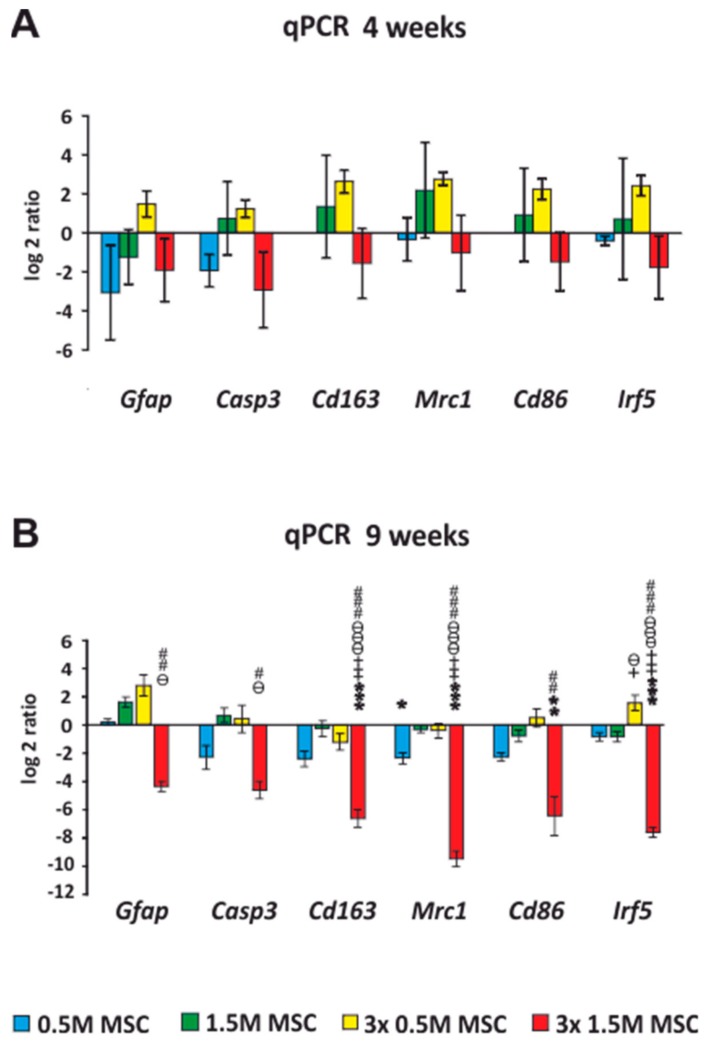
mRNA expression of selected genes 4 and 9 weeks after WJ-MSCs transplantation into the SCI. The graphs show the log2-fold changes of the ΔΔCt values of the indicated genes in comparison to the animals treated with the saline, which were set to 0 and are represented as x axis in the graphs. The expression of genes, which are related to the M1 (*Irf5*, *Cd86)* and M2 (*Mrc1*, *Cd163*) macrophage phenotypes, astrogliosis (*Gfap*), and apoptosis (*Casp3*) are shown 4 weeks after the SCI (**A**) and 9 weeks after the SCI (**B**). All of them were significantly downregulated in the group treated by 3 × 1.5 M and remained stable throughout the whole experiment. Data are expressed as mean ± SEM. * *p*  <  0.05 versus saline; ** *p*  <  0.01 versus saline; *** *p*  <  0.001 versus saline; + *p*  <  0.05 versus 0.5 M MSCs; ++ *p*  <  0.01 versus 0.5 M MSCs; +++ *p*  <  0.001 versus 0.5 M MSCs; # *p*  <  0.05 versus 3 × 0.5 M MSCs; ## *p*  <  0.01 versus 3 × 0.5 M MSCs; ### *p*  <  0.001 versus 3 × 0.5 M MSCs; ѳ *p*  <  0.05 versus 1.5 M MSCs; ѳѳ *p*  <  0.01 versus 1.5 M MSCs; ѳѳѳ *p*  <  0.001 versus 1.5 M MSCs. MSC: human Wharton Jelly mesenchymal stem cell; SCI: spinal cord injury; *Mrc1*: mannose receptor C type 1; *Casp3*: Caspase-3; *Gfap*: glial fibrillary acidic protein.

Samples from the spinal cord for qPCR analysis were taken 4 (Figure 3A) and 9 weeks (Figure 3B) after the cell transplantation. A comparison was made against the saline-treated rats, which were set as 0. The expression of genes, which are related to the M1 (*Irf5*, *Cd86*) and M2 (*Mrc1*, *Cd163*) macrophage phenotypes, astrogliosis (*Gfap*), and apoptosis (*Casp3*), was analyzed. Pro-inflammatory genes *Irf5* and *CD86* were, 4 weeks after the transplantation of the cells, insignificantly upregulated in the groups treated by 1.5 M and 3 × 0.5 M but, 9 weeks after the transplantation, were downregulated in all groups except the 3 × 0.5 M. Statistical significance against the saline group was found only in the group 3 × 1.5 M (*p* < 0.05).

The anti-inflammatory related genes *Mrc1* and *Cd163* were, 9 weeks after the transplantation, downregulated in all subjected groups with a significant difference in groups 1 × 0.5 M (*p* < 0.05) and 3 × 1.5 M (*p* < 0.001). However, groups treated by a total number of 1.5 M cells (1.5 M and 3 × 0.5 M) were, 4 weeks after transplantation, insignificantly upregulated; thus, their dynamics changed throughout the experiment.

Four weeks after the cell implantation, *Gfap*, except in the 3 × 0.5 M group, was downregulated in all groups; 9 weeks after the cell implantation, it remained downregulated only in the 3 × 1.5 M group, with a significant difference that corresponds with the immunohistochemical analysis of the astrogliosis that was lowest in this group. The expression of *Casp3* insignificantly decreased in the 0.5 M and 3 × 1.5 M groups and remained stable throughout the entire experiment.

(One-way ANOVA, Treatment; *Gfap* − H_4_ = 18.454, *p* = 0.001, *Casp3 −* H_4_ = 15.153, *p* = 0.004, *Cd163* − F = 20.243, *p* ˂ 0.001, *Mrc1* – F = 59.317, *p* ˂ 0.001, *Cd86* − H_4_ = 19.170, *p* ˂ 0.001, *Irf5* − F = 47.495, *p* ˂ 0.001; for *p* values of post hoc pair-to-pair test, see Supplement 4).

### 2.5. Cell Survival

Survival of the transplanted cells (0.5 M and 1.5 M) was evaluated by staining with the antibody against HuNu, two weeks after the transplantation. Surviving cells were detected as green clusters. Most of the cells remained at the site of the implantation—caught between the folds of arachnoidea mater in the cauda equinae. There was, however, a difference in the number of cells present. While after 0.5 M application only a few cells were detected, the application of 1.5 M resulted in a greater number of trapped cells. No homing into the lesion site was observed.

## 3. Discussion

The present study aimed to determine the effect and optimal dosage of transplanted stem cells derived from Wharton’s jelly into injured spinal cord. Cultured human WJ-MSCs were intrathecally implanted into spinal cord compression lesions of Wistar rats, and the impact on the recovery of the spinal cord tissue was described.

We compared single and triple repeated intrathecal delivery of hWJ-MSCs with a different number of cells (0.5 M and 1.5 M) in each application. Since cell survival in the vertebral canal is rather low [20,22,23], we hypothesized that, by repeated application, the trophic and immunomodulatory effect of the cells can be substantially increased. The route of MSC delivery varies in previous studies, and local or systemic approaches have been proposed and investigated, but the optimal method of delivery has yet to be determined.

We have chosen intrathecal delivery in our experiments. It eliminates the risk of direct surgical implantation without the need for deep analgesia and anaesthesia for the animal, and yet still guarantees a wide dissemination of cells through the subarachnoid space and around the lesion site [24]. The procedure can be done either by lumbar puncture or by suboccipital puncture of the cisterna magna. In our study, we chose lumbar puncture because it is more relevant to clinical medicine, where, especially in adult neurosurgery, it is an everyday occurrence. Additionally, repeated application via lumbar puncture is a lesser burden for animals than a suboccipital puncture of the cisterna magna. Since cell survival in the vertebral canal is rather low (approximately 14 days), repeated application substantially increased the trophic and immunomodulatory effect of the cells and could even be feasible in human patients.

Despite reports showing that intralesional transplantation provides better accumulation of the effector cells in the injury site [25], meta-analyses did not prove any statistical significant differences in locomotor improvement after delivery of MSCs using intravenous, intraparenchymal, and intrathecal administration [15,26]. Moreover, Pal et al. compared hBM-MSC injection into the spinal cord parenchyma and application via lumbar puncture. They reported that, while both i.t. and intraspinal transplantation showed improvement in histological evaluation, only the i.t.-transplanted group performed better in all behavioral tests [27]. Recently intranasal delivery of BM-MSCs with their successful migration into the spinal cord canal was reported, but compared to direct intrathecal delivery, there was significantly lower number of cells detected and the locomotor recovery was insignificant [28].

Besides the determination of transplantation route, optimal timing of transplantation is one of the most important factors to be considered. The subacute phase, defined as the time period between 3 and 14 days post-SCI in rodents and 2 months in humans showed better cell survival as a result of the reduced aggressive host environment due to the inflammation response, and when compared to the acute phase and unlike the chronic phase, the glial scar formation has not yet been formed [18,29,30]. Therefore, we had cell application start 7 days after lesion induction. Another disputable question remains regarding the appropriate dosage of transplanted cells. The vast majority of studies proving the effect of MSCs on SCI have focused only on single transplantation of 0.2–1 M of cells [31,32,33,34,35]. Alternatively, repetition and higher dosage is considered to be superior to single delivery. Li et al. compared single, triple, and quintuple transplantations of 1 M BM-MSCs and concluded that triple delivery is the best [36]. Similar results were found by Cizkova et al. with the conclusion that high doses and/or repetition of the transplantation may lead to higher efficacy of cell survival, more engraftment into the host tissue, and an improvement in functional outcome.

No such experimental study with hWJ-MSCs has been conducted, so evidence of the effect of single vs. multiple deliveries, together with various amounts of cells, is in high demand.

Analysis of behavioral tests revealed that functional recovery of hind limb motion in treated rats was partially dependent on the number of applied cells, which is in agreement with findings by Himes et al., who used a similar number of hBM-MSCs [32]. Whereas the single dose of 0.5 M hWJ-MSCs showed no significant difference in BBB testing, other groups achieved similar significantly higher scores, regardless of repeated application. However, in the advanced motor function testing, such as crossing the beam, the effect was visible in animals with repeated injections. The dose-dependent effect of hBM-MSCs in more advanced motor tests (grid walk and inclined plane test) was also observed by Pal et al. who compared doses of 2 M and 5 M cells/kg body weight [27]. No effect was detected in the rotarod test, which requires a higher level of motor coordination and stepping. Our experimental setting was comparable to our previous study with the hBM-MSCs [20]. However, application of 0.5 M hBM-MSCs resulted in functional improvement similar to the one observed with single application of 1.5 M hWJ-MSCs. It is generally accepted that, due to the low ability of MSCs to survive in the donor tissue, as confirmed in our study, and their limited in vivo differentiation, the main therapeutic effect of MSCs lies in their ability to secrete trophic factors promoting local neovascularization, inhibiting cell death, and suppressing the immune response [37]. We did not perform any in vitro analysis of growth factors and bioactive molecules, which would compare the paracrine effect of hWJ-MSCs and hBM-MSCs. Though various studies include the analysis of secretory factors, results are not unified in terms of the strength of secretion of regeneration-associated neurotrophic factors. Balasubramanian et al. found that WJ-derived cells secreted higher levels of neurotrophic factors bFGF, NGF, NT3, NT4, and GDNF compared to BM and adipose tissue (AT)-derived cells [38]. Hsieh and colleagues compared MSCs derived from Wharton’s jelly and bone marrow regarding their ability to regenerate infarcted myocardia; they described secretome differences that make Wharton’s jelly-derived MSCs a more angiogenic, neuroprotective, and neurogenic option [39]. On the other hand, Amable et al. reported that WJ-MSCs secreted very low concentrations of VEGF-4.070 and 4.614 times lower than BM- and adipose tissue MSCs (AT-MSCs), respectively [10]. Most studies agree that hWJ-MSCs have a more effective immunosuppressive function compared to hBM-MSCs [40,41]. However, all those studies were performed in vitro, where the proliferation rate of the WJ-MSCs (cell doubling time 40 h) is much higher than the hBM-MSC doubling time of 70 h [42], resulting in more paracrine effective cells in the medium and thus higher overall secretome. Our results confirmed relatively short population doubling time of hWJ-MSCs, which was approximately 34 h compared to 84 h of hBM-MSCs (see Supplement 1, Figure S1). Another possible explanation of the lesser effect of the same number of transplanted cells in our study is the smaller size of WJ-MSCs when compared with BM-MSCs, which can result in higher dilution in the host environment, or even leakage through the canal after the lumbar puncture. On the other hand, due to a smaller size, more cells can fit into small volumes and these cells can more easily spread through the CSF in the canal. Therefore, the dose-dependent effect can be more visible in the case of hWJ-MSCs than in hBM-MSCs.

Immunohistochemical analyses showed that transplantation of hWJ-MSCs facilitates axonal sprouting and plays a role in decreasing glial scar formation. Astrogliosis, which is closely bound up with reactive astrocytes was significantly less present in cell-treated rats. Axonal sprouting was increasingly enhanced as the total number of transplanted cells increased. This is in agreement with the findings of Li et al., who described the effect of WJ-MSCs in SCI as mainly due to the decreased expression of interleukin-1β (IL-1β) and the increased expression of nerve growth factor (NGF), which plays an important role in axonal growth [43]. The same trend was present in the measuring of the white and gray matter preservation, which revealed a strong neuroprotective effect in gray matter, mainly in the center of the lesion and only in groups with the highest number of treated cells. A significant effect on tissue sparing was observed only in animals treated by single or triple implantation of 1.5 M hWJ-MSCs. All these finding are in contrast with behavioral analysis, where no differences between higher dosage cell-treated animal groups were found in the BBB test. It is evident that, for the simple locomotor test, such as the BBB test, a threshold of a minimum number of cells is required to trigger functional improvement. However, closer analyses of tissue microstructure have shown that an increased number of cells applied repeatedly further improve the neuroprotective and neuroregenerative properties of the hWJ-MSCs.

Overall, our results proved a substantial benefit from treatment of SCI with MSCs and are in many ways comparable to other studies that used human BM-MSCs, AT-MSCs, or UC-MSCs in rats [27,32,35,44,45,46,47,48,49] or dogs [50,51]. Meta-analysis from 2014 by Oliveri confirmed the beneficial effect of MSCs in traumatic spinal cord injury but could not detect any clear association between locomotor recovery and MSCs isolated from specific tissues [15]. Still, because of the relative easy harvesting of hWJ-MSCs from otherwise discarded tissue, we believe that hWJ-MSCs are the most suitable for potential wide use in clinics.

Most of the clinical studies with mesenchymal stromal stem cells were performed on chronic lesions with total paraplegia (Frankel A, AIS A), lasting months after the primary injury [52]. Despite some reports that note some neurological improvement after transplantation [53], the effect of WJ-MSCs in the chronic model of SCI must be elucidated. However, we suggest that this treatment should be proposed for patients with spinal trauma that causes “only” compression of the spinal cord, which is visible in the magnetic resonance imaging (MRI) of T2 and STIR sequences as a hyper intense area of the spinal cord—myelopathia. These patients usually have preserved residual motor and sensory functions (Frankel B and above) and have a good prognosis of recovery when the best medical therapy is applied. hWJ-MSCs, since they are allogenic, can be easily up-scaled, prepared in advance, cryopreserved, and made ready for use in a relatively short time. Therefore, repeated application of hWJ-MSCs could add another piece to the yet unsolvable puzzle of how to treat SCI.

## 4. Materials and Methods

### 4.1. Cell Culture

The collection and isolation of WJ-MSCs were performed by explant culture according to the methodology described by Koci et al. [54]. In brief, human WJ-MSCs were obtained from discarded human umbilical cords from healthy full-term neonates after spontaneous delivery, with the informed consent of the donors using the guidelines approved by the Institutional Committee at University Hospital (Pilsen, Czech Republic). About 10–15 cm per umbilical cord were aseptically transported into sterile phosphate buffered saline (PBS; IKEM, Prague, Czech Republic) with antibiotic-antimycotic solution (Sigma, St. Louis, MO, USA) at 4 °C. After the removal of blood vessels, the remaining tissue was cut into small pieces (cca 1 mm^3^) and put into Nunc culture dishes (Schoeller, Thermo Fisher Scientific, Waltham, MA, USA) containing the alpha-Minimum Essential Medium (αMEM; East Port, Prague, Czech Republic), supplemented with 5% platelet lysate (IKEM, Prague, Czech Republic) and gentamicin 10 mg/mL (Sandoz, Holzkirchen, Germany), and cultivated at 37 °C in a humidified atmosphere containing 5% CO_2_. After 10 days, the explants were removed from the culture dishes, and the remaining adherent cells were cultured for 3 weeks or until 90% confluence and passaged using 0.05% Trypsin/EDTA (Life Technologies, Carlsbad, CA, USA). After passaging, the cells were seeded into culture flasks (Nunc; Schoeller, Thermo Fisher Scientific, Waltham, MA, USA) at a density of 5 × 10^3^ cells/cm^2^. The medium was changed twice a week. Cells of the 3rd passage were characterized by flow cytometry and their growth properties and differentiation potential was assessed (see Supplementary Material 1). For the transplantation, cells in the 3rd passage were used.

### 4.2. Animals

As an experimental model, adult Wistar male rats were used. Animals were obtained from the breeding facility of the Academy of Sciences of the Czech Republic. All experiments were performed in accordance with the European Communities Council Directive of 22nd of September 2010 (2010/63/EU) regarding the use of animals in research and were approved 8 September 2014 by the Professional Committee for Laboratory Animal Welfare of the Institute of Experimental Medicine, Academy of Sciences of the Czech Republic in Prague as the experimental project number 53/2014. All animals were approximately 10 weeks old, with weight varying between 275–305 g (*n* = 90). The first group of animals (*n* = 47), surviving 9 weeks, was used for behavioral testing, histological, immunohistochemical, and qPCR analysis (9W). The second group of animals (*n* = 39) was used for qPCR evaluation 4 weeks (4W) after SCI and was not included in behavioral testing. An additional four animals in the two groups (*n* = 4) were used for evaluation of surviving cells two weeks after transplantation of 0.5 and 1.5 M hWJ-MSCs.

### 4.3. Spinal Cord Injury Model

The surgical procedure was performed in an operating theatre under standard conditions. As a model of SCI, a balloon-induced ischemic-compression lesion was used [21,55]. At the beginning of the surgery, the animals were anesthetized with Isoflurane (Forane; Abbott Laboratories, Queenborough, UK), analgesia was induced by intramuscular injection of carprofen (Rimadyl, Cymedica, Horovice, Czech Republic, 4 mg/kg), and surgical prophylaxis was maintained by intramuscular injection gentamicin sulfate (Sandoz, Holzkirchen, Germany 5 mg/kg). After the skin incision, the paravertebral muscles were separated at the level of thoracic vertebra T7–T12 and laminectomy of T 10 was performed. A sterile 2-french Fogarty catheter was carefully inserted into the epidural space until the center of the balloon rested on the level of thoracic vertebra 8 (T8). The balloon was rapidly inflated with 15 μL saline and kept for 5 min. During this procedure, 3% isoflurane in air was administered at a flow rate of 0.3 L/min, and the animal’s body temperature was kept at 37 °C with a heating pad. After 5 min, the catheter was rapidly deflated and removed, and separated muscles and incised skin were sutured by single non-absorbable stitches. The lesioned animals were assisted in feeding and urination until they had recovered sufficiently to perform these functions on their own. The animals received gentamicin sulfate (5 mg/kg) for 7 days to prevent postoperative infections and were allowed to feed and drink ad libitum.

### 4.4. Transplantation

Transplantation of hWJ-MSCs was performed on the 7th, 14th, and 21st day after the SCI. Treatment was given intrathecally by a lumbar puncture between L3 and L4 or L4 and L5 through a 25 G needle under the short-time general anaesthesia described above. Firstly, a small volume of CSF was taped as a proof of the subarachnoid space. During the injection of the saline with hWJ-MSCs there was a movement of the rats tail as a response to irritation of the nerve roots of cauda equina. After injection the needle was rested in situ for 30 s to prevent backflow of the content. Animals were divided into 5 groups with variable treatment. The first group (**0.5 M**) received a single transplantation of 0.5 M hWJ-MSCs in 50 μL saline on the 7th day after SCI (*n* = 12). The second group (**1.5 M**) received a single transplantation of 1.5 M hWJ-MSCs in 50 μL saline on the 7th day after SCI (*n* = 9). The third group (**3 × 0.5 M**) received a triple transplantation of 0.5 M hWJ-MSCs in 50 μL saline on the 7th, 14th, and 21st day after SCI (*n* = 8). The fourth group **(3 × 1.5 M**) received a triple transplantation of 1.5 M hWJ-MSCs in 50 μL saline on the 7th, 14th, and 21st day after SCI (*n* = 7). The control group (**saline**) received a single or triple injection of 50 μL saline on the 7th (14th and 21st) day after SCI (*n* = 11). No differences between simple and repetitive transplantation of the saline were found, so the results were pooled together as a single control when compared with other types of treatment. The day before the transplantation, all animals received cyclosporine A by an intraperitoneal injection (10 mg/kg), which continued daily until the end of the experiment.

### 4.5. Behavioral Analysis

#### 4.5.1. BBB Test

The BBB open field test, originally described by Basso, Beattie, and Bresnaham [56] was used to assess the joint movement, weight support, forelimb-hindlimb coordination, paw placement and stability of the body. The rats were placed on the floor surrounded by boundaries making a rectangular shape. Results were evaluated in the range of 0–21 points: 0 indicated complete lack of motor capability and 21 indicated the best possible score (healthy rat). Measurements were performed every week for 8 weeks starting the first week after SCI.

#### 4.5.2. Rotarod Test

A rotarod unit machine (Ugo Basile, Comerio, Italy) was used to test the advanced degree of motor coordination of the limbs according to the method first described by Dunham and Miya [57]. Ability to balance on a rotating rod was recorded. Each animal was taught this task one week before surgery. Animals were placed on a rotating rod at a fixed speed of 10 rpm before surgery and 5 rpm after surgery and were left to walk for 60 s. There were four trials per day within five consecutive days. Between trials there was always a 5 min break. The latency to fall off the rod onto the floor was measured.

#### 4.5.3. Beam Walk Test

In the flat beam test, we tested the ability to cross a 1 m long narrow beam with a flat surface. Rats were placed on one side of the beam, and on the other side an escape box was placed. The latency and the trajectory to traverse the beam were recorded by a video tracking system (TSE-Systems Inc., Bad Homburg, Germany) for a maximum of 60 s. Performance of locomotor coordination was evaluated using a 0–7 point scale modified from Metz and Whishaw [58].

### 4.6. Histological and Immunohistochemical Analyses

At the end of the experiment (9 weeks after the SCI), all animals were anesthetized with ketamine (100 mg/kg) and xylazine (20 mg/kg) and transcardially perfused with a phosphate buffer solution (250 mL), followed by a 4% paraformaldehyde solution in a phosphate buffer (250 mL). The spinal cord was dissected and removed from the spinal column and embedded in paraffin wax. Serial cross sections (5 μm thick) were obtained by microtome within a 2-cm-long segment around the center of the lesion. Samples of five animals from each group were analyzed for the total volume of spared white and gray matter, axonal sprouting, and the extent of glial scar. Sections were stained with Luxol fast blue and Cresyl violet to distinguish the white and gray matter, with anti-GAP43 antibody to evaluate axonal sprouting or by anti-GFAP primary antibody to visualize the glial scar and reactive astrocytes.

#### 4.6.1. Cresyl Violet-Luxol Staining

**Figure 4:**
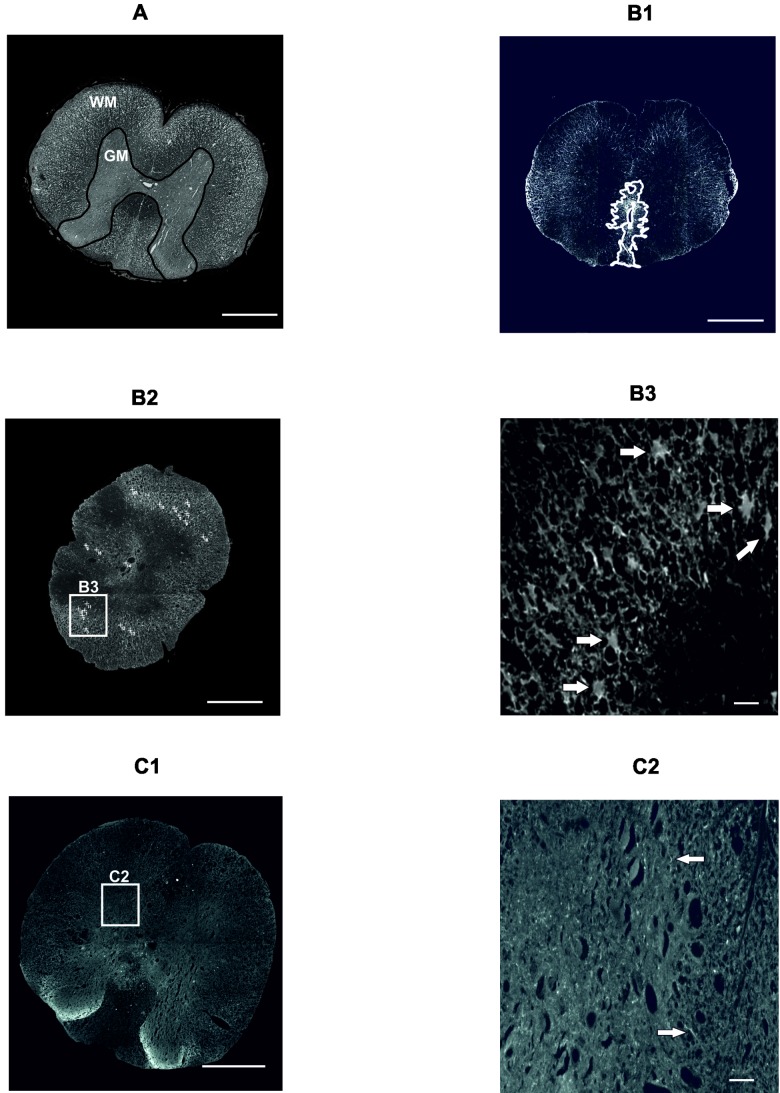
Illustrative images of morphometric and immunohistochemical analyses 9 weeks after SCI. Microscopic image of a section stained with Cresyl violet and Luxol fast blue to distinguish the white (WM) and gray (GM) matter (**A**). Scale bar: 500 μm. The marked glial scar around the main cavity (**B1**) and the total number of protoplasmatic astrocytes (**B2**) with a detailed inset (**B3**) from slices stained with GFAP-CY3. Scale bars: 500 μm (**B1**,**B2**), 10 μm (**B3**). Illustrative image of a section labeled with a GAP43^+^ antibody (**C1**) with a detailed inset (**C2**). Arrows point at GAP43^+^ fibers, which were manually counted as described in the Methods section. Scale bars: 500 μm (**C1**), 20 μm (**C2**). SCI: spinal cord injury; GM: gray matter; WM: white matter; GFAP-CY3: glial fibrillary acidic protein cyanine 3; GAP43: growth-associated protein 43.

For visualizing white and gray matter of the spinal cord, Cresyl violet and Luxol fast blue staining were used. Samples from five animals from each group were obtained. Each sample of the spinal cord was cut at 1 mm intervals. A total number of 15 cross sections, including the center of the lesion, were observed with an Axioskop 2 plus microscope (Zeiss, Oberkochen, Germany). Acquired images were analyzed for the total area of spared gray and white matter by ImageJ software (NIH, Bethesda, MD, USA) (Figure 4A).

#### 4.6.2. GFAP Staining

To determine the extent of the glial scar, immunohistochemical analysis of a CY3-conjugated primary antibody against GFAP (Sigma, St. Louis, MO, USA) was used. Samples from five animals from each group were obtained. Each sample of the spinal cord was cut at 1 mm intervals. A total number of 15 cross sections, including the center of the lesion, was observed with an Axioskop 2 plus microscope (Zeiss, Oberkochen, Germany). The acquired images were analyzed using ImageJ software (NIH, Bethesda, MD, USA). The GFAP positive area around the central cavity (Figure 4(B1)) together with the number of protoplasmic astrocytes (Figure 4(B2,B3)) was measured on each section.

#### 4.6.3. GAP43 Staining

The newly sprouted axons were visualized immunohistochemically using a primary antibody against GAP43 (Millipore, Billerica, MA, USA). Samples from five animals from each group were obtained. Each sample of the spinal cord was cut at 1 mm intervals. A total number of 15 cross sections, including the center of the lesion, were observed with an Axioskop 2 plus microscope (Zeiss, Oberkochen, Germany). The acquired images were analyzed and the number of GAP43-positive fibers per section was manually counted (Figure 4(C1,C2)).

### 4.7. qRT-PCR

To evaluate the up- or downregulation of expression rat target genes (*Gfap*, *Mrc1*, *Irf5*, *Cd163*, *Cd86*, *Casp3*) the quantitative real-time reverse transcription polymerase chain reaction (qRT-PCR) was used. Expression was evaluated at 4 and 9 weeks after hWJ-MSC administration (five animals from each group). Studied RNA was isolated from the paraffin cross sections of injured spinal cord around the center of the lesion using the High Pure RNA Paraffin Kit (Roche, Penzberg, Germany) following the manufacturer’s recommendations. The amount of RNA was measured by a spectrophotometer (NanoPhotometerTM P-Class, Munchen, Germany). cDNA was obtained from the isolated RNA by a reverse transcription using the Transcriptor Universal cDNA Master (Roche), and a thermal cycler (T100™ Thermal Cycler, Bio-Rad, Hercules, CA, USA). The qPCR chain reactions were performed using cDNA solution, FastStart Universal Probe Master (Roche, Penzberg, Germany) and the following TagMan^®^ Gene Expression Assays (Life Technologies): Casp3/Rn00563902_m1, Gfap/Rn00566603_m1, Cd86/Rn00571654_m1, Irf5/Rn01500522_m1, Mrc1/Rn01487342_m1, and Cd163/Rn01492519_m1.

The final solution of 25 ng extracted RNA diluted in 10 μL of solution was amplified on a StepOnePlus™ real-time PCR cycler (StepOnePlus™, Life Technologies, Carlsbad, CA, USA). The process of amplification was repeatedly performed under the same standard conditions: 120 s at 50 °C, 300 s at 95 °C, followed by 40 cycles of 15 s at 95 °C and 60 s at 60 °C. Each array included also a negative control (water). As a reference gene, Gapdh was used. The ΔΔ*C*t method was used for relative quantification of gene expression. All results were analyzed with StepOnePlus^®^ software (StepOnePlus™, Life Technologies, Carlsbad, CA, USA). Differences between the transplanted and control (saline) groups were analyzed for statistical significance with Δ*C*t values level, using a one-way ANOVA test with a post hoc pair-to-pair test. Differences were considered statistically significant if *p* < 0.05. Data are expressed as the mean ± the standard error of mean. The values of saline-treated animals were set as zero.

### 4.8. Statistical Analyses

Presented graphs with obtained data are shown as the mean ± SEM. The statistical significance between the groups treated by WJ-MSCs and the saline (control) group was assessed using either one-way ANOVA, or two-way ANOVA in the case of a second factor (time). Differences between the groups in behavioral tests and in areal measuring of the gray/white matter sparing and GFAP positive area of glial scar were assessed by two-way repeated measurement (RM) ANOVA. The Student–Newman–Keuls (SNK) post hoc pair-to-pair test was used to specify for which groups and at which time points the changes were significant. Statistical evaluation of the expression of the rat target genes was performed with one-way ANOVA test with a post hoc pair-to-pair test. In case of non-parametric values Kruskal–Wallis one-way ANOVA on ranks test was used. (All in Sigmastat 3.1, Sistat Software Inc., San Jose, CA, USA). Differences were considered statistically significant if *p* < 0.05.

## 5. Conclusions

Implantation of human MSCs derived from Wharton’s jelly improves functional outcome, by modulating the inflammatory response, inducing axonal sprouting and remodeling the glial scar. The effect is dose-dependent, with the best result achieved by repeated delivery.

## Supplementary Materials

Supplementary materials can be found at http://www.mdpi.com/1422-0067/19/5/1503/s1.
