# Supplementary material for: The Effect of Human Mesenchymal Stem Cells Derived from Wharton’s Jelly in Spinal Cord Injury Treatment Is Dose-Dependent and Can Be Facilitated by Repeated Application
Source: Int J Mol Sci. 2018 May 17;19(5):1503. doi: 10.3390/ijms19051503 (PMC5983761; doi:10.3390/ijms19051503)
Supplement: Supplementary file 1 [file ijms-19-01503-s001.zip › ijms-296577 supplementary/Krupa_Supplement 1.pdf]

Supplement 1

Human Wharton´s jelly mesenchymal stromal cells characterization

Methods

Human Wharton´s jelly mesenchymal stromal cells (hWJ-MSCs) were isolated using explant culture, according to the methodology described by Koci et al. [54].

As suggested by Brudet et al [1] and Greenwood et al. [2], cell proliferation was assessed in population doublings (PD). At each passage (P), population doubling time (PDT; 1) PD; (2) was calculated according to these formulas:

$$\text{PDT} = (t_2\text{-}t_1)\ .\ \frac{\log(2)}{\log\left(\frac{number\ of\ cells\ at\ t2}{numberof\ cells\ at\ t1}\right)} \quad (1)$$

$$\text{PD} = \frac{\log(numberof\ harvested\ cells) - \log(number\ of\ seeded\ cells)}{\log 2} \quad (2)$$

Cumulative PD (cPD) was determined as the PD at each passage added to the population doubling of the previous passage (Fig. 1).

hWJ-MSCs in the 3rd passage were analyzed for surface marker expression and multipotent differentiation potential.

Flow cytometry was performed using FACSAria^{TM}(Becton Dickinson, San Jose, California) to confirm the mesenchymal phenotype of hWJ-MSCs. The following antibodies against human antigens CD14, CD34, CD45, CD73, CD90, (Exbio, Czech Republic), CD105 (BioLegend, San Diego, CA, USA), HLA ABC, and HLA-DR (Pharmingen, USA) were tested. Data analysis was performed using BD FASCDiVa software.

hWJ-MSCs were differentiated into adipocytes, osteoblasts and chondrocytes to show their multilineage potential as described in [3]. Cell differentiation into various lineages was confirmed by specific staining and observed under a light or fluorescent microscope.

Results

The average PDT time of hWJ-MSCs during the study period, from P1 to P10, was 33.94 ± 2.26 hours. hWJ-MSCs maintained similar PD between passages (Fig 1), which confirms their relative long-term stability and low senescence.

Supplementary Table 1: *Populationdoublingtime (PDT) in hours, and populationdoubling (PD) of WJ-MSCs at differentpassages*

hWJ-MSC PD										
PassageNb.	1P	2P	3P	4P	5P	6P	7P	8P	9P	10P
PDT (days)	1.23 ±0.15	1.14 ±0.14	1.06 ±0.14	1.01 ±0.13	1.40 ±0.19	1.68 ±0.17	1.61 ±0.15	1.75 ±0.18	1.84 ±0.04	2.08 ±0.04
PD (index)	3.37 ±0.31	3.99 ±0.40	3.71 ±0.22	4.13 ±0.23	4.13 ±0.32	4.23 ±0.30	3.50 ±0.37	3.81 ±0.29	3.54 ±0.04	3.63 ±0.02

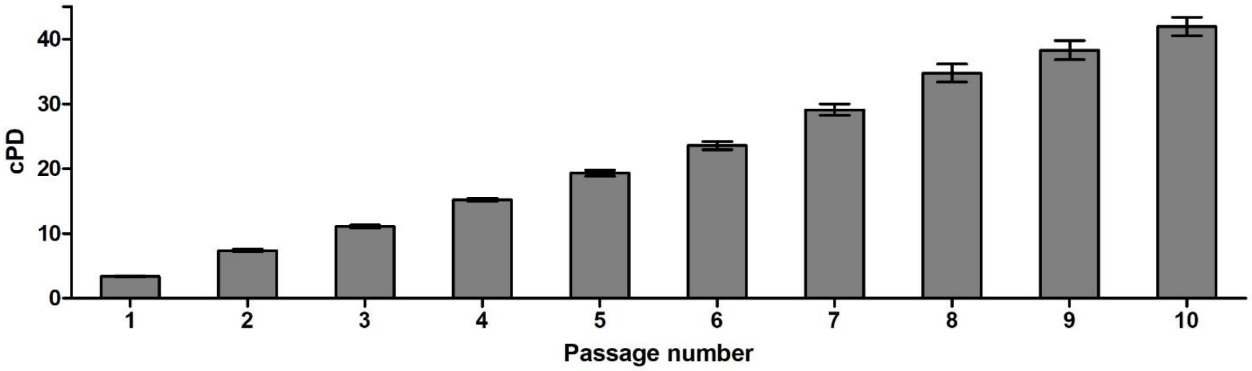

Fig 1. cumulative population doubling (cPD) of hWJ-MSCs in different passages. Data are shown as mean ± standard error mean, n = 7.

The mesenchymal phenotype of hWJ-MSCs was confirmed by the presence of CD29, CD73, CD90, CD105 and HLA-ABC and lack of CD14, CD31, CD34, CD45 and HLA-DR (Fig. 2).

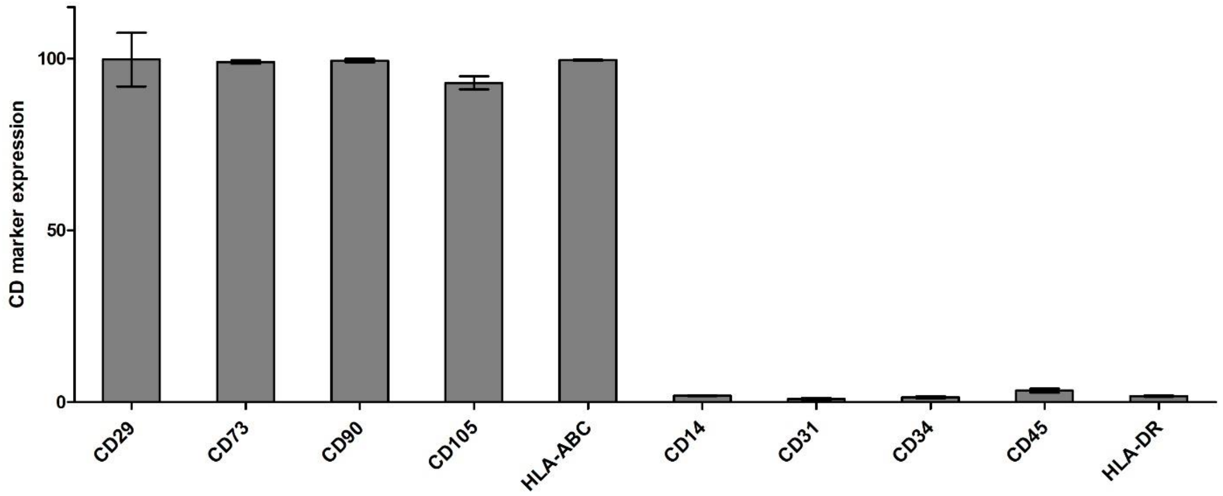

Fig. 2. Flow cytometry analysis of the surface markers of hWJ-MSCs in the 3rd passage. Data are shown as mean ± standard error mean, n = 7.

hWJ-MSCs at the 3rd passage were differentiated into all three cell-lineages: adipocytes, osteoblasts, chondrocytes (Fig 3). Adipocytes were detected by positive Oil-Red-O staining for lipid droplets. Osteoblasts were detected by positive Alizarin Red S staining for calcium-rich deposits. Chondrocytes were detected by Alcian blue staining for acid mucopolysaccharides.

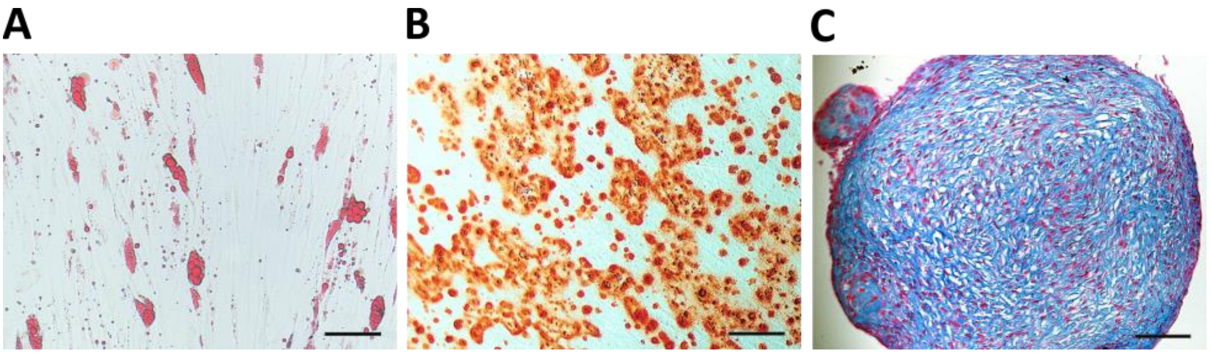

Fig.3. Multipotent differentiation of human Wharton jelly mesenchymal stromal cells (hWJ-MSCs) Differentiation into (A) adipocytes, (B) osteoblasts, and (C) chondrocytes. The scale represents 100 µm.

1. **Bruder, S.P., N. Jaiswal, and S.E. Haynesworth, *Growth kinetics, self-renewal, and the osteogenic potential of purified human mesenchymal stem cells during extensive subcultivation and following cryopreservation.* J Cell Biochem, 1997. 64(2): p. 278-94.**
2. **Greenwood, S.K., et al., *Population doubling: a simple and more accurate estimation of cell growth suppression in the in vitro assay for chromosomal aberrations that reduces irrelevant positive results.* Environ Mol Mutagen, 2004. 43(1): p. 36-44.**
3. **Koci, Z., et al., *Characterization of human adipose tissue-derived stromal cells isolated from diabetic patient's distal limbs with critical ischemia.* Cell Biochem Funct, 2014. 32(7): p. 597-604.**

54. **Koci, Z., et al., Extracellular Matrix Hydrogel Derived from Human Umbilical Cord as a Scaffold for Neural Tissue Repair and Its Comparison with Extracellular Matrix from Porcine Tissues. Tissue Eng Part C Methods, 2017. 23(6): p. 333-345.**
